# A Steep Learning Curve: Decoding Epigenetic Influence on Behavior and Mental Health

**DOI:** 10.1289/ehp.120-a396

**Published:** 2012-10-01

**Authors:** Bob Weinhold

**Affiliations:** Bob Weinhold, MA, has covered environmental health issues for numerous outlets since 1996. He is a member of the Society of Environmental Journalists.


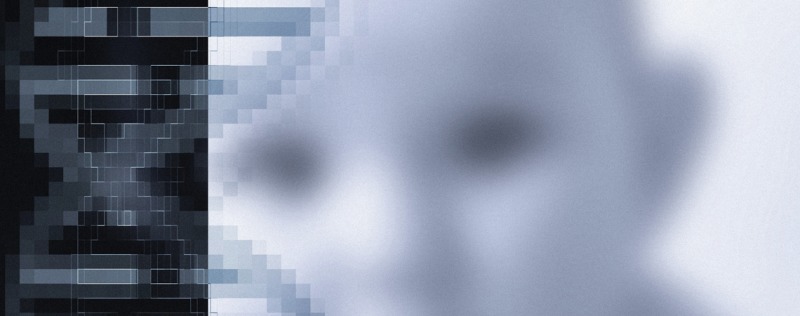
Research on epigenetics has surged in the past two decades as it has become apparent that changes in gene function aside from those related to DNA mutations or natural variations may be integral factors in numerous perplexing health disorders. But much remains unknown about this relatively new field. Of the thousands of epigenetics studies published,^1^ a few hundred have addressed behavioral and mental health outcomes, but only a fraction of those have dealt with fetal or childhood exposures or outcomes. However, early results in the niche field of behavioral epigenetics suggest such studies could provide insights into behavioral and mental health conditions such as autism spectrum disorders (ASDs), attention deficit/hyperactivity disorder, schizophrenia, bipolar disorder, depression, and anxiety.

## Examining Endocrine Disruptors

Studies of certain substances—including a variety of metals, solvents, polycyclic aromatic hydrocarbons (PAHs), particulate matter, pesticides, polychlorinated biphenyls (PCBs), and more^2^^,^^3^^,^^4^^,^^5^—have indicated they may trigger epigenetic changes of one kind or another. Frederica Perera, director of the Columbia Center for Children’s Environmental Health at Columbia University, and her colleagues have investigated several chemicals for potential effects on neurodevelopmental outcomes, with a focus on endocrine disruption that may involve epigenetic pathways.^3^ “The ability of chemicals to disrupt endocrine systems is a red flag for potential risk,” she says. “Epigenetics is being considered an important mechanism through which various stressors can operate. Many lines of evidence point to their being a special concern during rapid fetal development.”

Bisphenol A (BPA), an endocrine-disrupting compound used in many plastics and resins, has been assessed in a few behavioral studies. Published evidence, mainly from nonbrain tissues, indicates BPA may have the ability to affect the epigenome, leading to long-lasting changes in gene expression.^6^

At least one animal study suggests that prenatal BPA exposure may affect social behavior and brain gene expression and that the effects may extend to future generations.^7^ Although the authors of this study hypothesize that epigenetic mechanisms may be involved, they did not investigate epigenetic changes induced by BPA. However, they did examine associations across multiple generations, exposing dams to BPA before and during pregnancy at concentrations relevant to typical human exposure, then analyzing behavioral outcomes extending out to the fourth generation.

Both males and females in the generation exposed *in utero* engaged in less social behavior than nonexposed offspring, but mice in the fourth generation of the exposed lines—which had not been directly exposed to BPA—engaged in increased social behaviors (such as side-by-side interactions with a partner). The brains of the fourth-generation males from exposed lines had lower gene transcription levels for oxytocin, and the same was true for vasopressin for fourth-generation males and females from exposed lines. Each of these hormones is an estrogen target gene known to play a role in behavioral manifestations.

Effects across generations were also observed in a study of the endocrine-disrupting fungicide vinclozolin.^8^ Following exposure of pregnant female rats to high concentrations of the compound, the males and females of the unexposed third generation displayed opposing behaviors: Females had an increase in anxiety-like behavior, and males had a decrease in such behavior along with an increase in hyperactivity.

The researchers also found evidence of transgenerational epigenetic effects in a number of genes that have been associated with conditions such as schizophrenia, ASDs, depression, drug abuse, and social dysfunction in animals and humans. There were distinct differences between males and females in the number and type of affected genes in the hippocampus and amygdala, and in neural pathways such as mitogen-activated protein kinase signaling, neuroactive ligand-receptor interaction, axon guidance, long-term potentiation, and gonadotropin-releasing hormone signaling. The authors say they can’t make specific correlations between genes, pathways, and brain functions, but note there have been observations generally linking disrupted programming of brain gene expression and altered behavior.

## Other Paths to Explore

Psychoactive drugs (both therapeutic and recreational) are being investigated for potential epigenetic effects. Only one animal study has provided direct evidence of maternal exposure to a psychoactive drug and epigenetic changes in the brains of offspring, according to Marija Kundakovic, a postdoctoral research scientist and lecturer at Columbia University’s Department of Psychology. In that study, DNA methylation was significantly altered in mouse offspring exposed prenatally to cocaine, compared with unexposed offspring.^9^ For other drugs, including antidepressants and methamphetamine,^10^ correlations have been seen in humans between fetal exposures and neurobehavioral outcomes, although such outcomes have not been definitively linked to epigenetic mechanisms; indeed, many other mechanisms are possible.

Diet may also play an important role in behavioral epigenetics. In one study, researchers fed pregnant rats a diet that was deficient in methyl donors (substances such as folic acid that can transfer methyl compounds to other substances), then assessed the offspring for changes in hippocampal DNA methylation and gene expression as well as associated behavioral changes in female pups as they aged.^11^ The methyl donor–deficient diet contained 90% less choline, folate, and methionine than the control diet. Pups were fed regular chow after weaning.

The researchers investigated methylation changes at selected sites in four genes involved in stress or memory pathways with previous evidence indicating that expression is related to DNA methylation of the gene. The glucocorticoid receptor (*Nr3c1*) and 11β-hydroxysteroid dehydrogenase type 2 (*Hsd11*β*2*) are involved in glucocorticoid signaling and metabolism, respectively; neuronatin (*Nnat*) is a paternally expressed imprinted gene^12^ present in the brain and shown to be involved in brain development; and *reelin* codes a protein known to be involved in the regulation of neuronal migration in the developing brain and in learning and memory processes.

In adulthood, females born to nutrient-deficient mothers were more adaptable to changing environments (indicating better learning skills) but showed increased anxiety compared with controls. The combination of these two traits is consistent with evidence that increased, moderate anxiety improves learning.

In general, there was no difference in expression of the four genes evaluated, with the exception of *reelin* among females in one of two age groups tested. They found four CpG sites (DNA regions where cytosine and guanine are paired) with significantly different methylation of 40 evaluated. However, the one gene that showed a difference in expression—*reelin*—did not show a significant difference in methylation, and the researchers conclude that they can’t explain the learning and anxiety differences solely by the changes in the four genes investigated.

At the same time, the nutrients studied play a role in many different physiological processes, and potential health effects of deficiencies could arise through nonepigenetic mechanisms. The results also could have been affected by the fact that the nutrient-deficient mothers ate much less and ended up weighing 18% less than controls, with one day longer gestation and a significantly lower number of pups per litter. Their pups also had a much higher number of newborn deaths and very different sex ratios from controls. It took a full year for the pups of nutrient-deficient mothers to catch up to the weight of the controls. These confounding factors may have skewed the results.

## The Stress Connection

Other forces such as stressful early-life events have been associated with evidence of epigenetic changes and with behavioral and mental health outcomes that might be partly related to epigenetic effects. For instance, researchers have observed a correlation between increased childhood stress and increased adulthood methylation in a region of the promoter of the glucocorticoid receptor gene (*NR3C1*) in leukocyte DNA.^13^ This was based on a study of 99 healthy adults who reported childhood exposure to stressors such as parental death or desertion, abuse or neglect by the parent, or parental detachment, coldness, or rejection. Elevated glucocorticoids have been linked with outcomes such as impaired neuronal growth, modified immune function, and accelerated cellular aging. These outcomes have also been associated with early-life stress and major depression in several studies.

The more categories of stress that participants reported, the greater the levels of methylation in the genes examined. In turn, there was a notable link between increased methylation of *NR3C1* and weakened cortisol response to a standardized neuroendocrine challenge test that is designed to evaluate aspects of neurotransmission function. That result fits with a common observation that prolonged stress can initially contribute to elevated cortisol levels but eventually lead to reduced levels.

The researchers adjusted for potential confounders such as age, sex, use of estrogens (e.g., oral contraceptives), body mass index, and selected childhood socioeconomic factors. However, they had limited data on a number of factors that could play a role in the observed methylation changes, such as hypothalamic–pituitary–adrenal axis function. They investigated only a small number of people for a limited number of pathways, and they acknowledge more research is needed to confirm and elucidate many aspects of their findings. But their findings suggest epigenetic mechanisms may help explain the oft-identified links between stressful childhood experiences and adverse mental health outcomes in adulthood. The findings are consistent with a number of similar animal and human studies.^14^

In a behavioral analysis study, investigators examined 25 healthy young men who had been followed at ages 6–15 years and evaluated during that time by teachers for levels of physical aggression.^15^ Based on analysis of blood T cells when the children were older, those with more adolescent aggression had significantly higher *SLC6A4* methylation at two CpG sites than those with low or no aggression. For blood monocytes, the researchers found the same relationship for two other CpG sites, and, on average, for all 24 CpG sites investigated. They also found a link for three of the sites between higher mean methylation and lower serotonin synthesis in the orbitofrontal cortex of the brain, which is known to play a role in decision making. This finding is consistent with other studies of links between *SLC6A4* and serotonin synthesis. However, there are factors and processes other than *SLC6A4* involved in serotonin synthesis, so this study alone doesn’t prove a link.

The researchers selected blood cells to work with since it’s virtually impossible to evaluate brain cells in living humans, a problem common to much research in this field. Yet gene expression varies between different types of white blood cells, and some of this variation could derive from epigenetic mechanisms. There is extensive debate over what constitutes suitable biomarkers and where in the body to find them. Other researchers have suggested sources such as buccal or placental cells, the latter being favorable as it plays numerous critical functional roles during gestation that can influence neurodevelopment.^16^^,^^17^

## Looking for a Cure

Understanding the roles of genetics or the environment alone or interactions of the two has not led to extensive success in treating mental health or behavioral disorders, as many had hoped.^18^ Some experts in the field predict that adding the epigenetic perspective will significantly boost the odds of success.

**Figure:**
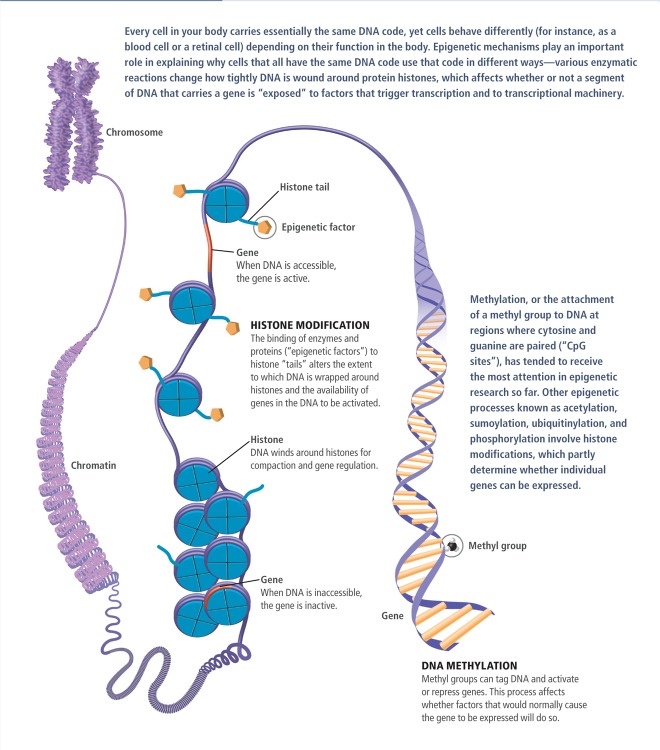
Basic Concepts in Epigenetics Epigenetics is a relatively new field, and much is unknown. It is believed that some changes can be passed down through generations. Other epigenetic changes are transient, and in terms of environmental influences on health it is too soon to say which type of change may be most important. © 2012 janewhitney.com

“Epigenetic changes are potentially reversible and [may be] preventable if research findings are used to inform public health recommendations and policies,” Kundakovic says. However, although there are indeed examples of epigenetic changes being reversible, the exact mechanisms through which this process occurs remain subject to many unknowns and variations and the topic of great debate.^19^

Indeed, says Barry Lester, a professor of psychiatry and pediatrics at Brown University and Women and Infants’ Hospital, clinical applications aren’t likely very soon. “I get concerned when people talk about treatment and prevention,” he says. “We don’t even know the role of epigenetics in normal development. The groundwork that needs to be done is substantial.”

Some investigators have proposed that certain pharmaceuticals^20^^,^^21^ may someday prove useful in addressing mood and behavior disorders through epigenetic means. However, these drugs typically affect many body processes, making it difficult to target a narrow pathway and minimize adverse side effects, and often their mechanisms of action remain poorly understood. For many foods and nutrients, there is some evidence of epigenetic effects, both beneficial and adverse. Among the foods and nutrients with such evidence are biotin, cruciferous vegetables (e.g., broccoli), flavonoids, garlic, genistein, green tea, iron, isoflavones, lipoic acid, lycopene, polyphenols, potassium, selenium, turmeric, vitamins A, B_1_, and E, wine, and zinc.^22^^,^^23^ And exercise and psychotherapy also have been proposed as potentially beneficial modes of therapy, once much more information is available.^24^^,^^25^

**Figure:**
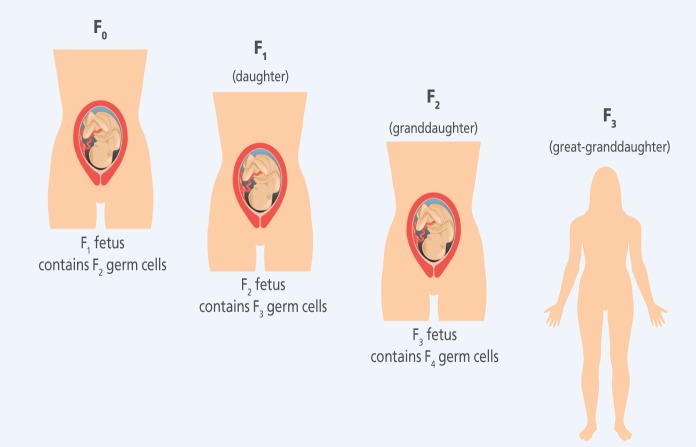
Transgenerational Exposures When a pregnant woman (F_0_) is exposed to an agent, there is also direct exposure to her fetus (F_1_) and to the second successive generation (F_2_) that exists as developing germ cells within the fetus. The first woman’s great-granddaughter—the F_3_ generation—represents the first generation with no direct exposure to the original agent.

There’s quite a bit of science investigating another potential therapeutic approach: A number of studies have explored epigenetic links between positive behavior in rat offspring whose mothers were more nurturing during the lactational period.^14^ In one of the more recent studies, researchers evaluated male rat offspring for correlations between degree of maternal licking/grooming and expression and methylation of *GAD1*, the gene encoding a glutamic acid decarboxylase enzyme. Alterations in *GAD1* expression have been linked with disorders such as schizophrenia.^26^

Based on analysis of hippocampal tissue, the investigators found *GAD1* expression was significantly elevated at postnatal day 4 and in adulthood in pups that received high levels of licking and grooming as neonates. In contrast, the methylation of *GAD1* promoter sites was significantly higher in the pups who had received low grooming and licking, and *GAD1* expression in those pups was significantly lower in adulthood. The results add to the growing evidence that nurturing during childhood may affect adult mental health, although much remains unknown about the specifics of this relationship.

## Lots of Homework to Be Done

Before people can benefit from treatment and prevention, a number of core issues need to be addressed. For instance, basic baseline information is lacking since much of the existing research focuses on aberrant situations, such as exposure to toxics or a behavioral disorder. Little is known about what normal epigenomes look like.^16^

Research in animals remains tricky because factors as basic as housing conditions, handling regimes, light cycles, and social contacts may modify the epigenome, and there is little in the way of standardized protocols to minimize these effects.^16^ In humans, with their far less controlled environments, such influences tend to be much more problematic, to say the least.

More research in humans is essential, says Carmen Marsit, an assistant professor of pharmacology and toxicology at Dartmouth University. “The epidemiological community has been slower in picking up on this work and moving into human populations,” he says. “Part of the problem there is addressing the question of where to look. In animals, brains are readily available. This is not possible in true population-based studies, so some creativity and some novel insights are needed.”

Marsit, Padbury, and Lester have published studies relating epigenetic changes in placental genes to neurobehavior in the newborn.^1^^,^^22^ “These are studies of normal infants that are critical because they could mean that there are baseline [relationships] between epigenetic changes and neurobehavior that we have to know about before we know what is deviant or what abnormal changes are,” Lester says.

Another major limitation is that only a few genes have been studied, leaving thousands of others still to be investigated. Kundakovic points out that more widespread use of the epigenomic approach—studying epigenetic changes across the whole genome—will enable investigators to determine how many and which genes are modified in response to a particular environmental exposure, then validate such changes in a different study population. “While this method is less sensitive than a single-gene approach, it can allow the discovery of important new candidate genes,” she says. This kind of work likely will be most effective if researchers do as Padbury notes he and others have done: team up with other disciplines to better understand complex interactions.

To make the most efficient use of funds and focus and expedite work as effectively as possible, Lester suggests approaches such as multidisciplinary projects similar to those sponsored by U.S. National Institutes of Health program project grants, or new epigenetics research institutes funded by multiple sources and/or joint public–private funding. In any case, he says, “it will take a massive, intensive, coordinated effort that needs direction.”
